# Does growing up at high altitude pose a risk factor for type 2 diabetes?

**DOI:** 10.3934/publichealth.2019.1.96

**Published:** 2019-03-06

**Authors:** Martin Burtscher, Hannes Gatterer, Johannes Burtscher

**Affiliations:** 1 Department of Sport Science, University of Innsbruck, Austria; 2 Austrian Society for Alpine and Mountain Medicine, Austria; 3 Institute of Mountain Emergency Medicine, EURAC Research, Bolzano, Italy; 4 Laboratory of molecular and chemical biology of neurodegeneration, École Polytechnique Fédérale de Lausanne (EPFL), Switzerland

Type 2 diabetes mellitus (T2DM) has developed into a serious and one of the largest global health problems. Whether growing up and/or living in high-altitude regions pose a risk factor for T2DM remains a matter of debate. A potential reason for the conflicting findings is the challenge of disentangling the role of hypoxia among a plethora of other factors (like age, sex, body mass, physical activity, diet, smoking, alcohol drinking, etc.).

## 1. T2DM risk in Argentinean children living at high altitude

Recently, Hirschler and colleagues reported in this journal a higher T2DM risk in indigenous Argentinean children living at 3750 meters when compared to children living at 1400 meters [1]. This is in agreement to others, showing a link between the oxy-hemoglobin saturation at altitude and the development of the metabolic syndrome and T2DM [2].

## 2. Findings from other high-altitude regions

The findings reported by Hirschler et al. are in contrast to a large-scale study among 285,196 US adults living at high altitude (1500–3500 m) who had a lower adjusted chance of having diabetes than people living close to sea level. The odds ratio (95% CI) for diabetes was 0.95 (0.90–1.01) between 500 and 1,499 m, and 0.88 (0.81–0.96) between 1500 and 3500 m compared to low altitude (0 to 499 m) [3]. Importantly, these authors also demonstrated an inverse relation between altitude and obesity, and better glucose control at higher altitudes [3]–[5]. Another study using the Finnish Diabetes Risc Score (FINDRISC) questionnaire was recently performed in Kyrgyzstan and also found that the 10-year risk of T2DM development was greater in residents living at low altitude (500–1200 m) compared to those at high altitude (2000–4500 m) [6]. Conversely, one study performed in high and low altitude regions of Peru actually suggests a greater incidence of T2DM at high compared to low altitude. Importantly, T2DM there was largely attributed to obesity [7]. The association between obesity and T2DM even in children is well known [8].

## 3. Insulin resistance is not impaired in Argentinean children living at high altitude

In the study of Hirschler et al., children living at lower altitude had significantly higher body mass and body mass index compared to those at high altitude [1]. Thus, the question arises, why children living at high altitude should be at greater risk for development T2DM. Systemic blood pressure, cholesterol (total and LDL) and glucose levels were higher in high-altitude children but, at least based on the mean values (SD), these levels were normal and did not indicate any adverse effects of the high-altitude condition. For instance, the mean systolic blood pressure was 87 (±14) mmHg [1] compared to 100 mmHg as the 50^th^ percentile of systolic blood pressure in the same age group of a reference population [9]. Moreover, it should be kept in mind that higher blood pressure values might simply be related to lower temperature at high altitude (6.5 °C decrease per 1000 m) due to cutaneous vasoconstriction as recently pointed out [10]. Beside the reported metabolic risk factors, insulin resistance (IR) is one among the most important predictors for T2DM. Since Hirschler et al. determined both insulin and glucose levels, the homeostasis model assessment of IR (HOMA-IR) can be used to determine IR. A HOMA-IR value of 3.16 was proposed as cut-off, above which a significant IR may be present in adolescents [11]. Using the mean values presented in the paper of Hirschler et al., the HOMA-IR was 1.2 for the children living at high altitude and 1.4 for those at lower altitude, thus without any indication for an elevated T2DM risk neither in children living at high nor in children at low altitude.

Undoubtedly, Hirschler and colleagues provide highly interesting clinical and metabolic characteristics from a relatively large sample of schoolchildren from different elevations, but in our opinion, a higher T2DM risk cannot be derived from the presented findings.
